# 

**Published:** 1857-09

**Authors:** 


### LIST OF COLLABORATORS.

John L. Atlee, M.D., President Pennsylvania State Medical Society.
Walter F. Atlee, M.D., Philadelphia.
John Bell, M.D., Philadelphia, formerly Professor of Medicine, Medical College of Ohio.
T. S. Bell, M.D., Louisville, Ky.
S. M. Bemiss, M.D., Louisville, Ky.
L. P. Blackburn, M.D., Natchez, Miss.
G. C. Blackman, M.D., Professor of Surgery, Medical College of Ohio.
W. S. Bowen, M.D., New York.
J. L. Bobbs, M.D., formerly Professor of Anatomy, Indianapolis.
W. M. Boling, M.D., Montgomery, Ala., formerly Professor of Midwifery, Transylvania University, Lexington, Ky.
N. Bozeman, M.D., Montgomery, Ala.
J. T. Bradford, M.D., Augusta, Ky.
John H. Brinton, M.D., Lecturer on Operative Surgery, Philadelphia.
W. S. Chipley, M.D., Professor of Medicine, Transylvania University.
A. Clapp, M.D., New Albany, Ia.
D. B. Cliffe, M.D., Franklin, Tenn.
J. Da Costa, M.D., Lecturer on the Practice of Medicine, at the Philad. Med. Association.
M. Dupierris, M.D., Havana, Cuba.
W. F. Edgar, M.D., United States Army.
S. C. Farrar, M.D., Jackson, Miss.
C. S. Fenner, M.D., Memphis, Tenn.
Austin Flint, M.D., Professor of Clinical Medicine and Pathology, University of Buffalo.
Charles Fricke, M.D., Baltimore.
W. Y. Gadbury, M.D., Benton, Miss.
O. C. Gibbs, M.D., Chautauque County, New York.
Dr. J. P. Hall, Glasgow, Ky.
John Hardin, M.D., Professor of Obstetrics, Kentucky School of Medicine, Louisville.
Edward Hartshorne, M.D., One of the Surgeons to Wills Hospital for Diseases of the Eye, Philadelphia.

E. B. HASKINS, M.D., Clarksville, Tenn., late President of the Tennessee State Medical Society.

C. A. HENTZ, M.D., Marianna, Florida.

ADDINELL HEWSON, M.D., One of the Surgeons to Wills Hospital for Diseases of the Eye, Philadelphia.

SAMUEL L. HOLLINGSWORTH, M.D., late Editor of the Medical Examiner, Philadelphia.

WILLIAM A. HAMMOND, M.D., United States Army.

WILSON JEWELL, M.D., Philadelphia, President of the Quarantine and Sanitary Convention.

G. A. KUNKLER, M.D., Madison, Ia.

WM. V. KEATING, M.D., Lecturer on Obstetrics and the Diseases of Women, in the Philadelphia Medical Association.

BEN. LOGAN, M.D., Shreveport, La.

A. LOPEZ, M.D., Mobile, Ala., formerly President of the Alabama State Medical Society.

GEORGE R. MOREHOUSE, M.D., Philadelphia.

H. WEIR MITCHELL, M.D., Lecturer on Physiology at the Philad. Med. Association.

B. I. RAPHAEL, M.D., New York.

J. C. REEVE, M.D., Dayton, Ohio.

L. C. RIVES, M.D., formerly Professor of Midwifery, Medical College of Ohio.

J. LAWRENCE SMITH, M.D., Professor of Chemistry, University of Louisville.

W. C. SNEED, M.D., Frankfort, Ky., President Kentucky State Medical Society.

C. H. SPILMAN, M.D., Harrodsburg, Ky., late President of Kentucky State Medical Society.

GEO. SUTTON, M.D., Aurora, Ia.

W. L. SUTTON, M.D., Georgetown, Ky., formerly President Kentucky State Medical Society.

HORATIO R. STORER, M.D., One of the Physicians to the Boston Lying-in Hospital, Boston, Mass.

S. THOMPSON, M.D., Albion, Ill.

E. WILLIAMS, M.D., Cincinnati, Ohio.

### Bi-Monthly Bibliographical Record.

### AMERICAN.

Elements of Pathological Anatomy. By Samuel D. Gross, M.D. Third edition, modified and thoroughly revised. Illustrated by three hundred and forty-two engravings on wood. 8vo. pp. 771. Republished by Blanchard & Lea, Philadelphia, 1857.

On Dysentery and its Treatment. By Henry Tiedemann, M.D., of Philadelphia. 12 mo. pp. 29. Philadelphia, 1857.

Therapeutic Cultivation; its Errors and its Reformation: an Address delivered to the Tennessee Medical Society, April 7th, 1857. By E. B. Haskins, M.D., President. Pp. 28. Nashville, 1857.

An Address delivered before the Medical Society of the State of Pennsylvania, at its Annual Session, held in West Chester, in May, 1857. By R. La Roche, M.D., President of the Society. 8vo. pp. 51. Philadelphia, 1857.

An Address on the Life and Character of the late Professor, John Lockey, delivered at the request of the Cincinnati Medical Society. By M. B. Wright, M.D. 8vo. pp. 72. Cincinnati, 1857.

Early History of the Operation of Ligature of the Common Carotid Artery. By James R. Wood, M.D., Surgeon to Bellevue Hospital, President of the New York Pathological Society, &c. Reprint. 8vo. pp. 58. New York, 1857.

An Inaugural Thesis on Intra-Capsular Fractures of the Cervix Femoris. By John Geo. Johnson. Pamphlet. Reprint, pp. 32. 8vo. New York.

### FOREIGN.

A Guide to the Treatment of Diseases of the Skin; with Suggestions for their Prevention: for the use of the Student and General Practitioner. By Thomas Hunt, F.R.C.S., &c. 2d edition. London, 1857.

Lives of British Physicians; Second edition, with the following lives added: Dr. Merriman, Dr. Paris, Dr. Halford, Dr. Chambers, and Dr. Clutterbuck. 8vo. pp. 416. London, 1857.

How to work with the Microscope. By Lionel Beale, M.B., F.R.S. London, 1857.

The Medical Profession. By Edwin Lee, M.D., &c. 8vo. London, 1857.

The Baths of Germany, France, and Switzerland. Two volumes in one. Third edition. By Edwin Lee, M.D. London.

Baths of Rhenish Germany. By Edwin Lee, M.D. London.

The Effect of Climate on Tuberculous Disease. (A Prize Essay.) By Edwin Lee, M.D. London.

The Structure and Functions of the Eye, Illustrative of the Power, Wisdom, and Goodness of God. By Spencer Thomson, M D. Illustrated by nearly one hundred engravings, London, 1857.

Hugman on Hip-Joint Disease: with reference especially to Treatment, by Mechanical means for the Relief of Contraction and Deformity of the Affected Limb. By Wm. Curtis Hugman, F.R.C.S., &c. London, 1857. 8vo.

Advice to Officers in India. By John M'Cosh, M.D., late of the Bengal Medical Staff. London, 1856.

Hygienic Medical and Surgical Hints for Young Officers of the Royal, and of the Merchant Navy. By W. M. Saunders, M.D., Surgeon, Royal Navy. London, 1856.

An Introduction to Clinical Medicine. By J. Hughes Bennett, M D., F.R.S.E., &c. Third edition. Edinburgh, 1857.

The Constitution of Women as Illustrated by Abdominal Cellulitis, or Inflammation of the Cellular Membrane of the Abdomen and Pelvis. By Charles Bell, M.D., &c. Edinburgh, 1857.

Traité Pratique des Maladies de la Peau, Par Alph Devergie, Médecin de l'Hôpital Saint Louis, &c. Deuxième édition. 8vo. Paris, 1857.

Guérison Radicale de la Goutte. Par le Docteur G. Bosel de Mens, Médecin adjonct de l'Hôtel Dieu, &c. Paris, 1857.

La Revue Pharmaceutique de 1856. Supplement à l'officine pour 1857, Par Dorvault, Pharmacien, Directeur Fondateur de la Pharmacie Centrale des Pharmacien de France. Paris.

Lehrbuch der Histologie des Menschen und der Thiere. Von Dr. Franz. Leydig. Frankfurt, 1857.

